# The hepatocellular model of fatty liver disease: from current imaging diagnostics to innovative proteomics technologies

**DOI:** 10.3389/fmed.2025.1513598

**Published:** 2025-03-05

**Authors:** Renee Hernandez, Natasha S. Garcia-Rodriguez, Marco A. Arriaga, Ricardo Perez, Auwal A. Bala, Ana C. Leandro, Vince P. Diego, Marcio Almeida, Jason G. Parsons, Eron G. Manusov, Jacob A. Galan

**Affiliations:** ^1^Division of Human Genetics, School of Medicine, The University of Texas Rio Grande Valley, Brownsville, TX, United States; ^2^South Texas Diabetes and Obesity Institute, School of Medicine, The University of Texas Rio Grande Valley, Brownsville, TX, United States

**Keywords:** MASLD, MASH, proteomics, exposomics, single-cell, proteogenomics

## Abstract

Metabolic Dysfunction-Associated Steatotic Liver Disease (MASLD) is a prevalent chronic liver condition characterized by lipid accumulation and inflammation, often progressing to severe liver damage. We aim to review the pathophysiology, diagnostics, and clinical care of MASLD, and review highlights of advances in proteomic technologies. Recent advances in proteomics technologies have improved the identification of novel biomarkers and therapeutic targets, offering insight into the molecular mechanisms underlying MASLD progression. We focus on the application of mass spectrometry-based proteomics including single cell proteomics, proteogenomics, extracellular vesicle (EV-omics), and exposomics for biomarker discovery, emphasizing the potential of blood-based panels for noninvasive diagnosis and personalized medicine. Future research directions are presented to develop targeted therapies and improve clinical outcomes for MASLD patients.

## 1 Introduction

Metabolic Dysfunction-Associated Steatotic Liver Disease is a chronic illness characterized by fat accumulation in the liver, unrelated to alcohol consumption. MASLD is typically asymptomatic and involves fat accumulation in at least 5 percent of hepatocytes without hepatocellular damage (1, 2). However, when accompanied by inflammation, it can progress to Metabolic Dysfunction-Associated Steatohepatitis (MASH), which can further progress to fibrosis and cirrhosis. MASH is characterized by fibrosis, lobular inflammation, hepatocellular ballooning, and steatosis (1).

MASLD is influenced by obesity, insulin resistance, high cholesterol, sex, lifestyle, and genetic-by-environmental interactions. It is associated with several comorbidities, such as diabetes, dyslipidemia, cardiovascular disease, and chronic kidney disease (1, 3, 4). The risk of developing this disease increases with low physical activity and high-caloric diets rich in saturated free fatty acids, cholesterol, and fructose (5). On the other hand, increased intake of antioxidant vitamins and fat esterified with polyunsaturated FFAs are associated with decreased risk of MAFLD. In individuals with genetic predispositions, environmental factors, such as smoking, pesticides, and air pollution, can further increase the chance of developing MASLD (1).

MASLD affects approximately 32.4% of the global population, making it one of the leading causes of chronic liver disease (6, 7). Prevalence is exceptionally high in North America, primarily due to the high obesity rates (8). Among the North American population, the highest prevalence is found in Hispanics (63.7%), followed by non-Hispanic whites (56%) and non-Hispanic blacks (40%). In the Rio Grande Valley, a region in south Texas where 90% of the population is of Mexican origin, the prevalence of MASLD is 64 %. This economically disadvantaged region also experiences significant health disparities, with high rates of diabetes (32.5%) and obesity (55.5%) (2, 9).

Recent advances in proteomics technologies have changed our understanding of liver disease pathology, diagnosis, and treatment options (10). We aim to provide an overview of proteomics’ current and future applications in liver disease research and management. We will review the foundation of liver pathophysiology, diagnosis, and clinical care and explore how proteomics is critical in advancing the study of liver disease.

### 1.1 Pathophysiology, diagnostics, and clinical care for MASLD

#### 1.1.1 Pathophysiology

Triglyceride (TG) accumulation in hepatocytes drives the pathophysiology of MASLD (11). MASLD is driven by insulin resistance, which disrupts glucose and lipid metabolism, causing triglyceride (TG) accumulation in hepatocytes (12). Excess triglycerides in the liver result in lipotoxicity, the production of reactive oxygen species (ROS), inflammation, and hepatocyte failure, which ultimately leads to MASH (13). As the inflammatory disease progresses, fibrosis, cirrhosis, and hepatocellular carcinoma may develop, resulting in clinical manifestations of liver failure (13). The early stages of MASLD are characterized by histological abnormalities such as hepatic balloon degeneration, steatosis, lipid buildup, and inflammation (14). These changes result from the complex interplay between genetics, environmental factors, metabolic abnormalities, and their interactions (15). This multifactorial etiology of fatty liver disease (FLD) makes diagnosis, clinical trials, and treatment challenging (15).

#### 1.1.2 Diagnosis

Diagnostic techniques for liver disease are classified into invasive and noninvasive methods (Table 1). Noninvasive methods, such as blood-based analysis of hepatic enzymes (transaminases), are often used to screen for liver disease because they are cost-effective. Unfortunately, they are not valuable to exclude patients from liver biopsy (16) but have been incorporated into suggested biomarker panels (17). Other noninvasive measures of fibrosis include the Metabolic Dysfunction-Associated Fibrosis 5 score.MAF-5, MASLD fibrosis score (NFS), the fibrosis-4 index, and the AST (aspartate aminotransferase) to platelet ratio index.

**Table 1:** Existing diagnostic approaches to MASLD.

Diagnostic method	Description	Sensitivity/specificity	Cost	Pros/Cons	Ref
	Ultrasound: The primary imaging method to identify hepatic steatosis, effective when more than 33% of hepatocytes are steatotic. However, it is less reliable for mild steatosis.	Steatosis Stage1: 81%/92% Stage 2: 89%/70% Stage 3: 83%/63% Fibrosis Stage 1: 81%/77% Stage 2: 75%/82% Stage 3: 87%/89% Stage 4: 94%/91%	$200–$1.5 k	Pros - Rapid - Good diagnosis value - Increased performance with later disease stage Cons - High abdominal adiposity may impact scan performance	(137)
	FibroMeter Vibration Controlled Transient Elastography (VCTE): A noninvasive diagnostic tool that combines liver stiffness measurements obtained through vibration-controlled transient elastography with serum biomarkers from the FibroMeter panel, offering an accurate assessment of liver fibrosis.	Fibrosis Stage 2: 66.7%/86.4% Stage 3: 76.2%/81.3% Stage 4: 94.2%/70.4%	$200–$2000	Pros -Rapid -Noninvasive - Combination of Biomarkers and TE Cons -Cost -Availability - variability or inaccuracies in individual cases, particularly in patients with co-existing conditions affecting liver stiffness or biomarkers	(138, 139)
	Magnetic Resonance Elastography (MRE): MRE provides a more precise measurement of the amount of liver fat than magnetic resonance imaging (MRI). Notably, the performance of MRE is not dependent on the scanner’s magnetic field strength.	Steatosis 87.4%/74.3% Fibrosis 90.9%/82.9%	$400 k+	Pros - It can be economical to screen high-risk obese or diabetic populations. Cons - Cost - Accessibility	(140, 141)
Noninvasive Biochemical Scores	Metabolic dysfunction-associated fibrosis 5 score.MAF-5 Includes waist circumference, body mass index, diabetes, aspartate aminotransferase (AST), and platelet measures.	60.9% was predicted at low, 14.1% at intermediate, and 24.9% at high risk of fibrosis		Pros-validated, age-independent, anthropometric referral tool to identify individuals at high risk of liver fibrosis in primary care populations. Cons- Is used to identify not diagnose individual at high risk of hepatic fibrosis.	(142, 143)
	MASLD Liver Fat Score: This method uses metabolic syndrome parameters, fasting insulin, and liver enzymes to predict MASLD with high sensitivity and specificity.	N/A	~$150	Pros - The score is based on easily accessible clinical/laboratory parameters, easy to perform - Uses standard blood tests - Helps detect asymptomatic individuals Cons - May be less accurate in those with other causes of liver disease (e.g., viral hepatitis) - Not able to assess the severity of the disease	(144)
	Fibrosis-4 Index (FIB-4): integrates platelet count, AST, ALT, and age to determine the risk of fibrosis. Higher-risk patients may require further evaluation with transient elastography or enhanced liver fibrosis (ELF) tests.	N/A	~$150	Pros - Similar to Liver Fat Test (cost/blood) - Stratifies patients by liver fibrosis risk (low, intermediate, high) - High Negative Predictive Value rules out advanced fibrosis, reduces unnecessary additional testing Cons - Influenced by age - Less accurate for patients with intermediate scores often necessitates additional ELF tests	(145)
Invasive diagnostic techniques	Liver Biopsy: Liver biopsy is the gold standard for MASLD and MASH diagnosis and staging. It evaluates the degree of inflammation, fibrosis, hepatocyte damage, and steatosis. Liver biopsy is accurate, but its application is restricted to situations where noninvasive techniques are unsatisfactory. It is an invasive, expensive, and unsuitable procedure for all patients.	N/A	$3 k-$300 k	Pros - Gold standard in liver disease diagnosis - Low Cost (Most Locations) Cons - Medical facility required - Potential cost burden	(146)

Some diagnostic tools focus on microanatomy, including liver biopsy, Magnetic Resonance Imaging (with MRI-based MAST score) (18), ultrasound (19), and Vibration Controlled Transient Elastography (VCTE). VCTE (FibroScan) accurately measures steatosis and fibrosis and effectively assesses liver health in community settings (20–23). The FibroScan controlled attenuation parameter (CAP) measures steatosis by analyzing ultrasonic shear wave propagation through the liver, while the liver stiffness measurement (LSM) measures hepatic fibrosis. The FAST-AST score—a combined measurement of LSM, CAP, and AST—estimates the risk for progression to cirrhosis. A FAST score below 0.35 has a sensitivity of 90% to rule out cirrhosis, while a FAST score above 0.67 has a specificity of 90% for ruling it in (19).

Although noninvasive techniques are effective in providing an initial assessment of MASLD, the gold standard for diagnosis is an invasive liver biopsy, which is commonly associated with distress and discomfort (24).

#### 1.1.3 Clinical care

MASLD and early stages are managed with lifestyle modifications focusing on dietary changes, increased physical activity, and reduced alcohol intake (25). Advanced liver disease and fibrosis are managed with more intense lifestyle adjustments to achieve long-term weight loss (25). Depending on the severity, weight loss programs, medications for treating obesity, and bariatric surgery may also be advised (25, 26).

Currently, there are re-purposed pharmacologic and non-pharmacologic options for MASLD treatment and prevention, including thiazolidinedione (TZD) pioglitazone, glucagon-like peptide 1 receptor agonists (GLP-1), sodium-glucose cotransporter 2 inhibitors (SGLT2 inhibitors), vitamin E, flavonoids, and statins. Treatment targets triglyceride synthesis, metabolism imbalance, and free fatty acid production that contribute to MASLD. In a mouse model, flavonoids, such as *Chenopodium quinoa* Willd (CQWF), inhibit lipid accumulation by down-regulating the expression of two genes (CD36 and FASN) (27). Hesperitin, a flavonoid found in citrus fruits, influences DRP1, PINK1, and Parkin activity downregulating mitochondrial regulation dynamics (28).

Weight gain promotes genetic predisposition towards insulin resistance, resulting in excess lipolysis and flow of free fatty acids to the liver. This promotes intrahepatic triglyceride accumulation and, over time, may progress to steatohepatitis. Medications target reduced glucose, insulin secretion, lipid metabolism, weight loss, and hunger control and may reduce cardiovascular risk. Currently, Rezdiffra (resmethrin) is the only US Food and Drug Administration (FDA) approved treatment for noncirrhotic MASH with moderate to advanced fibrosis. Phase 3 trials are underway for other medications that target liver molecules. An excellent review of the targets for managing MASLD outlines the genes and proteins involved in the inflammatory pathways involved in fibrosis (29).

### 1.2 Future research for diagnosis and treatment

The utilization of biomarkers is becoming a viable method for noninvasive diagnosis of liver disease, monitoring of treatment response, and enabling personalized medicine. Identifying novel biomarkers for MASLD is crucial for advancing early disease diagnosis and improving clinical outcomes. Recent research has identified candidate serum biomarkers corresponding to each liver disease stage, including inflammatory cytokines, fatty acid transporter proteins, lipid droplet-associated proteins, and extracellular matrix proteins at various stages (30). Identifying these biomarkers can enhance the accuracy and speed of MASLD diagnosis, allowing physicians to develop patient-specific lifestyle and treatment plans (31).

Blood-based panels, such as NIS4^®^, have become important for identifying patients at risk of severe MASLD or MASH without the need for invasive procedures. Recent improvements, including the NIS2 + ™ panel, have shown a potential to further reduce unnecessary biopsies and screening costs in clinical practice and trials (32, 33). Neither test has yet received FDA approval.

Biomarkers also play a vital role in treating fibrosis. Fibroblast Growth Factor 21 (FGF21), a liver-secreted hormone regulating energy and lipid metabolism, shows promise as a therapeutic target. FGF21 analogs, such as efruxifermin and pegozafermin, are currently in clinical trials for treating fibrosis in MASLD (12). These biomarkers identify fibrosis, guide FGF21-based therapies, and monitor treatment responses, which can lead to more personalized and effective liver disease management.

Biomarkers provide insight into the molecular mechanisms of liver disease, helping clinicians assess key processes such as fat deposition, oxidative stress, inflammation, and fibrosis. Application in noninvasive diagnostics, genetic profiling, and blood-based panels enhance early detection and risk stratification. By predicting disease progression, particularly fibrosis, biomarkers allow for optimized treatment strategies and real-time monitoring of therapeutic responses to metabolic and antifibrotic agents. Biomarkers are also important for developing targeted therapies and serve as surrogate endpoints, reducing the need for liver biopsies and allowing for faster evaluation of treatment efficacy.

Current imaging technologies offer advantages and disadvantages (cost, accessibility, resolution of imaging, sensitivity, and specificity) to diagnose and manage MASLD. To address these gaps, we focus on methods to detect early-stage cellular events and changes in protein expression and identify key pathways involved in MASLD progression. Mass spectrometry-based proteomics offers promising opportunities for novel biomarker discovery and elucidating the molecular mechanisms underlying MASLD development (34, 35). Proteomic studies identify circulating protein signatures associated with MASLD progression. A comprehensive proteo-transcriptomic analysis of 4,730 circulating proteins in patients with MASLD identified distinct signatures for active steatohepatitis and advanced fibrosis (36). Biomarkers are the future of MASLD diagnosis, treatment, and research.

## 2 Mass spectrometry-based proteomics for MASLD biomarker discovery

Biomarkers are quantifiable proteins, metabolites, and genetic markers that indicate physiological states, pathological processes, or responses to therapeutic interventions. These biomolecules are typically extracted from various biofluids such as blood, cerebrospinal fluid (CSF), urine, saliva, and tissue samples. The data from biomarker analysis provide crucial insights for epidemiologists and clinicians, enabling informed decision-making in disease detection, diagnosis, prognosis, and treatment prediction (37–39). Blood plasma, a rich source of diverse proteins and metabolites, is pivotal in numerous biological processes. It offers significant advantages over a liquid biopsy due to its accessibility and minimal invasiveness compared to other sampling methods. The field of biomarker discovery has been propelled by the increasing feasibility of personalized medicine applications, allowing for real-time monitoring of disease states and therapeutic responses.

Blood and its derivative in plasma proteomics and metabolomics are obtained after centrifugation with anticoagulants (EDTA or heparin) and are prepared for analysis. Samples contain a complex mixture of proteins, lipids, metabolites, and other small molecules that can be analyzed and characterized using advanced mass spectrometry-based techniques. Such analyses are crucial for identifying and validating biomarkers for various conditions, including metabolic disorders, cancers, and neurodegenerative diseases (40). The blood plasma proteome has a dynamic range of 10 orders of magnitude, reflecting the individual’s physiological state. Unlike traditional biochemical diagnostics, current proteomic approaches simultaneously identify and quantify thousands of proteins, providing a comprehensive snapshot of health status. Post-translational modifications of proteins, often indicative of disease processes, can be detected and quantified. Proteomics offers unparalleled depth and breadth of analysis in biomarker discovery. High-resolution mass spectrometry, coupled with advanced bioinformatics tools, enables the detection of low-abundance proteins and subtle changes in protein expression levels that may indicate early-stage diseases or treatment responses. This level of detail is precious in developing multi-protein biomarker panels, which often provide greater specificity and sensitivity than single-protein markers (41). Protein, peptides, cleaved fragments, and their proteoforms (spliced variants, isoforms, post-translationally modified) can appear in the blood circulation from active secretion or cellular leakage, providing a window into the current state of human health. The demand for more translatable biological targets and the use of mass spectrometry is driving the need for more MS-blood-based proteomic studies. Plasma is a highly desirable bio-fluid for studying MASLD and identifying biomarkers because of the higher protein concentration and ability to detect proteins, metal ions, metabolites, lipids, and proteins.

Integrating omics methods with next-generation laboratory instrumentation and computational approaches represents the latest advancement in biomarker discovery. Identifying and quantifying proteins and peptides in proteomics have led to novel discoveries in the characterization of disease and health. More than 10,000 human proteins can be identified and quantified using proteomics, including low-abundant transcription factors present in cell and cell culture supernatants (42, 43). Incorporating proteomics in multi-omics analyses enhances high-throughput capabilities, facilitating the discovery of important proteins that may act as biomarkers for certain diseases. Unlike genetic-based biomarkers, protein-based approaches offer direct, targeted, highly sensitive, and specific quantitative analysis for biomarker discovery. Using noninvasive serum and plasma to identify disease-associated biomarkers, proteomic laboratories provide rapid sample processing and results with high specificity compared to conventional diagnostic methods (44, 45). These approaches are well suited for identifying and quantifying peptides, protein expression, and regulation associated with MASLD.

The value of collecting large-scale proteomics data in population studies provides opportunities to investigate non-genetic associations, capture biomarkers of environmental exposure, stratify individuals according to their state of health or disease, and monitor the longitudinal progression of disease. The development of large biobanks and population cohorts allows us to identify molecular phenotyping that can be performed across hundreds of thousands of individuals. These opportunities raise questions regarding which technologies to use, expected outcomes, and whether it is cost-effective to characterize the proteomes of entire populations.

### 2.1 Essential components for proteomics

**Figure 1:**
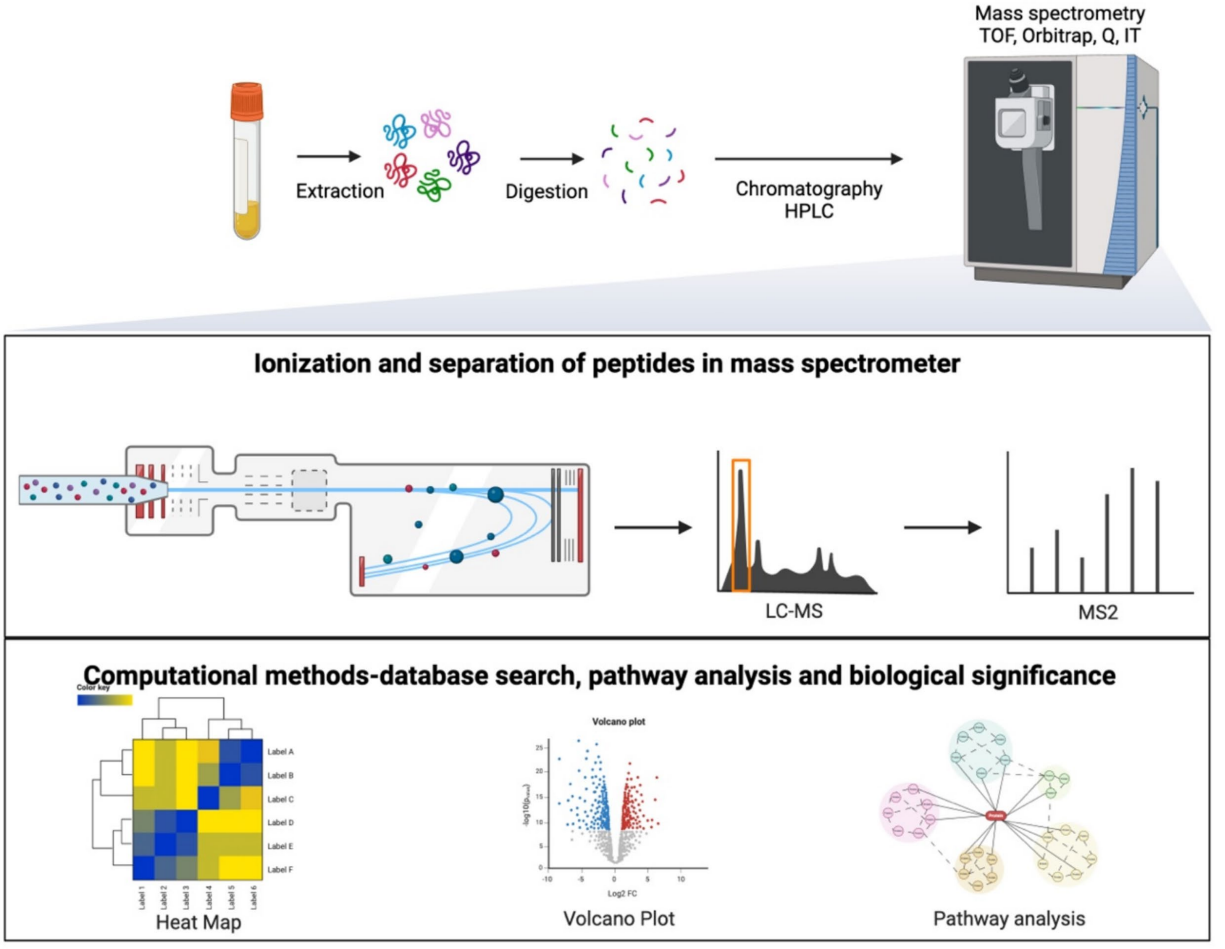
Proteomic workflow is used for sample preparation, mass spectrometry analysis, and bioinformatics.

Mass spectrometry is the gold standard for identifying and measuring proteins in proteomics, separating ions according to their mass-to-charge ratio (m/z), and enabling an evaluation of peptide masses. Tandem mass spectrometry (MS/MS) involves two stages of mass analyses with an intermediate fragmentation step in which nitrogen or helium gas fragments the ions into smaller masses for sequencing. Mass spectrometers are often coupled with High-Pressure Liquid Chromatography (HPLC), which separates peptides based on their interaction with a liquid mobile phase and a stationary phase. LC–MS/MS analysis offers an extremely selective and sensitive method for measuring peptides, as HPLC fractionation increases the number of peptide identifications (46). Spectra are collected in a 60-min run and stored as RAW files (uncompressed and unprocessed data). A single run can have over 12,000 spectra, requiring computational methods for database searching. These methods allow for identifying proteins by comparing the mass spectra to species-specific protein references (47, 48). One of the most widely used databases for this purpose is the UniProt Knowledgebase (UniProtKB). Pathway analysis can be applied to these large sets of identified proteins (Figure 1). This approach organizes large lists of proteins into smaller sets of proteins that function in the same pathways and biological processes (49). In the context of MASLD, annotated databases and pathway analyses can be utilized to identify specific proteins and pathways underlying this disease.

### 2.2 Advantages of proteomics over traditional methods in biomarker discovery

Traditional diagnostic methods often target a limited number of predefined validated markers and can underrepresent individuals from specific populations, comorbidities, ages, and health statuses. Proteomics offers several advantages for biomarker discovery, including improved accuracy, greater detection limits, depth of coverage for surrogate peptides, and broader applicability. One of the major advantages is global protein profiling, which makes it possible to analyze thousands of proteins in a biological sample at once (50). Another significant advantage includes elucidating post-translational modifications such as phosphorylation, glycosylation, acetylation, and ubiquitination. These modifications play crucial roles in disease processes and are not typically captured by traditional genomic or transcriptomic methods (51). Modern proteomic approaches facilitate quantifying protein expression across the proteome of both normal and diseased samples, providing valuable insights into protein composition, localization structure, function, interactions, and expression profiling (52).

## 3 From discovery of novel biomarkers to validation

The development of protein biomarkers is divided into discovery, verification, and validation. New protein biomarker candidates are identified without bias by using an untargeted approach and quantifying the global proteome, which provides a holistic and large pool of protein identifications/datasets that can be used to distinguish and classify biomarkers to differentiate disease from healthy individuals. The FDA-NIH Biomarker Working Group defines biomarkers as having the property that can be assessed to indicate normal biological processes, pathogenic processes, or responses to an intervention or exposure (53).

### 3.1 Validation of biomarkers

Biomarker validation involves testing in larger and more diverse cohorts to ensure they are statistically significant and clinically relevant. This phase involves clinical sample testing, in which biomarkers are validated using clinical samples from well-characterized patient cohorts, often including various stages of the disease, to assess their diagnostic and prognostic utility (54). Validation across multiple laboratories ensures that biomarkers are reproducible in various settings and populations, confirming that candidate biomarkers are vetted prior to clinical trials and eventual regulatory approval.

### 3.2 Novel biomarkers discovered through proteomics

#### 3.2.1 Diagnostic biomarkers

Diagnostic biomarkers are critical for early detection of diseases and can improve patient outcomes. In addition to MASLD and other conditions, several proteomic studies have identified promising diagnostic biomarkers such as Insulin-like growth factor-binding protein complex acid labile subunit (ALS) and Galectin-3 Binding Protein (Gal-3BP). These proteins are significant markers for distinguishing early-stage from advanced liver fibrosis in MASLD patients (55). Their plasma concentrations correlate with the degree of fibrosis, offering a noninvasive way to monitor the course of the illness and facilitate early identification.

#### 3.2.2 Prognostic biomarkers

Prognostic biomarkers provide clinicians with insight into how a disease is expected to progress, enabling them to forecast patient outcomes and adjust treatment regimens accordingly. Proteins such as IGFBP3/4 (Insulin-like Growth Factor Binding Proteins 3 and 4) and IGF-1 (Insulin-like Growth Factor 1) have been linked to the development of fibrosis in MASLD (56, 57). Their levels aid in long-term disease tracking and can be used to forecast the severity of fibrosis.

#### 3.2.3 Therapeutic targets

Therapeutic targets identified through proteomics are proteins that drugs can target to treat diseases. A protein panel called ADAMTSL2 (A Disintegrin and Metalloproteinase with Thrombospondin Motifs Like 2) was developed to distinguish between different phases of liver fibrosis in Metabolic Dysfunction-Associated Steatotic Liver Disease (58). This panel has demonstrated high accuracy in identifying advanced fibrosis stages, suggesting ADAMTSL2’s potential as a target for therapeutics.

## 4 Identification of MASLD biomarkers using proteomics

Specific examples of the advances in the use of proteomics and the identification of biomarkers encompass the role of biomarkers in MASLD research and management (31, 59–62), ranging from gene expression functional enrichment analysis to the role of inflammation in the development and pathogenesis of liver disease.

Proteomics databases are essential for comprehensive protein-related information, facilitating storing, organizing, and retrieving data on protein sequences, structures, post-translational modifications, and associated functional annotations. Integrating advanced proteomics technologies with these databases has significantly enhanced our understanding and management of MASLD. A pivotal study by Niu et al. (10) employed high-resolution mass spectrometry to profile the plasma proteome of 48 patients with varying degrees of MASLD and cirrhosis (63). This analysis identified six differentially expressed proteins: LDOB, APOM, LGALS3BP, PIGR, VTN, and AFM. Notably, AFM and LGALS3B had been previously implicated in liver disease by independent research groups (37, 39). The study also revealed a global clinical and proteomic data correlation map strongly associated DPP4, ANPEP, TGFBI, PIGR, and APOE with MASLD and cirrhosis progression. DPP4, ANPEP, and TGFBI emerged as potential therapeutic targets due to their correlation with liver enzymes secreted into plasma during hepatic injury (63).

Leveraging the Plasma Proteome database, serum proteome profiling has uncovered specific protein signatures related to MASLD progression (64). These signatures include proteins involved in immune system regulation and inflammation (e.g., RBP4), coagulation (e.g., fibrinogen *β* chain and fibrinogen *γ* chain), and extracellular matrix structure and function (e.g., Lumican). Additionally, carrier proteins in the blood, such as apolipoprotein C1, have shown potential in differentiating various liver disease conditions, thereby improving and enhancing diagnosis and prognosis. Xing et al. employed a sophisticated MS-based discovery-verification-validation proteomics workflow, combined with machine learning models, to identify a serum proteomic biomarker panel comprising HABP2, CD163, AFP, and PIVKA-II (65). This panel demonstrated the ability to distinguish early-stage hepatocellular carcinoma (HCC) from liver cirrhosis in healthy individuals, highlighting the potential of proteomic technologies in liquid biopsy applications for the early detection and management of liver diseases, including MASLD.

Utilizing the NCBI annotated database, liver tissue proteomics have revealed significant alterations in mitochondrial proteins, providing critical insights into hepatic disease pathogenesis (66). In an animal model of chronic ethanol exposure, 43 mitochondrial proteins exhibited differential expression, with 13 increasing and 30 decreasing. This study underscored the extensive impact of ethanol on the mitochondrial proteome and highlighted specific metabolic pathways involved in liver pathology. The Human Liver Proteome Project (HLPP) database represents a valuable resource for understanding the complete set of proteins expressed in the human liver (67). By focusing on proteome mapping, functional annotation, and clinical relevance, the HLPP database provides researchers with detailed proteomic data and analytical tools to advance the understanding and treatment of liver diseases, including MASLD.

Integrating proteomics databases with advanced analytical techniques has revolutionized our approach to MASLD research. These resources enable a deeper understanding of the molecular mechanisms underlying liver diseases, support the development of targeted therapies, and improve the potential for personalized treatment strategies. As we continue to harness the power of proteomics in MASLD research, we move closer to reducing this prevalent liver condition’s significant health and economic burden.

In addition, protein-based biomarkers, such as cytokines like TNF-*α* and Interleukin 6 (IL-6), have the potential to detect MASLD, suggesting that they can help identify inflammation-related Metabolic Dysfunction-Associated Steatohepatitis (MASH) and more advanced stages of fibrosis (68–71). An investigation conducted in both clinical and experimental settings has demonstrated the involvement of matrix metalloproteinases (MMPs) and their inhibitors, tissue inhibitors of metalloproteinases (TIMPs), in hepatic fibrogenesis and fibrinolysis (72, 73). In a retrospective study involving 84 patients with cirrhosis and 14 healthy controls, TIMP-1 levels in arterial and hepatic vein plasma were determined by using ELISA (74). The findings demonstrated a substantial positive correlation between TIMP-1 levels and the disease severity in patients with cirrhosis, suggesting that TIMP-1 may be a useful noninvasive marker for anticipating problems associated with cirrhosis. These biomarkers are essential indicators of fibrosis and play a pivotal role in evaluating disease progression and the risk of advancing to cirrhosis. Metabolomic biomarkers are also noteworthy, focusing on lipid profiles and metabolic disturbances that characterize MASLD. Another intriguing class of biomarkers includes microRNAs (miRNAs), such as miR-122 and miR-34a, which control inflammation, fibrogenesis, and liver metabolism (75). The noninvasive diagnostic potential of miRNAs’ allows for easy detection in blood samples, and their levels correlate strongly with liver pathology. Utilizing proteomics for the diagnosis of MASLD has the potential to make disease testing a regular part of annual screening. By using serum, high-throughput technologies enable rapid sample analysis and turnaround.

Recently, Amyloid beta (A𝜷) and associated amyloid precursor protein (APP) were found to protect against liver fibrosis. APP knockdown upregulates classical hallmarks of fibrosis. APP regulates mitochondrial function, lipid metabolism, and cell–cell interactions in a healthy liver, protecting against liver fibrosis. Further evaluation of the role of these specific proteins will provide valuable insight into diagnosis and treatment (76–78).

## 5 Emerging technologies

### 5.1 Extracellular vesicles (EV-omics)

The primary methods for diagnosing MASLD are histology-based. However, new diagnostic approaches are emerging and present a unique opportunity for monitoring disease progression in MASH stages, allowing for easily accessible noninvasive methods of identifying biomarkers (79–81). One such advancement involves using omics technologies to use extracellular vesicles (EVs) and exosomes as a noninvasive diagnostic approach to biomarker discovery.

EVs constitute a variety of membrane vesicles that are released from cells. They can be classified into apoptotic bodies, microvesicles (MVs), and exosomes (82). Apoptotic bodies are the largest EVs, 1,000–5,000 nm in size. They are released from apoptotic blebbing cells (external forms of the cells) that are undergoing cell death. MV’s range in size from 100 to 1,000 nm and are generated by external budding from the plasma membrane. Exosomes, the smallest of the EVs, have the smallest size of 30–100 nm and originate from the endosomal system (83). EVs are released into the extracellular space and deliver information to other cells. They also carry out essential cargoes used in cell communication (84). The cargo content comprises biologically important proteins, lipids, metabolites, and nucleic acids, making them critical targets in biological research (83, 85).

EVs can be explored in the pathological identification of rare diseases through cell communication’s cellular and molecular pathways, including homeostasis and other diseases such as cancer and neurodegenerative disorders (84, 86–88). EVs offer tremendous therapeutic opportunities in cancer, infectious diseases, and neurodegenerative disorders, including the potential to serve as important biologicals in drug delivery to targeted sites (89, 90). EVs also play direct roles as pathogenic elements, particularly in neurodegenerative disorders, cancer, and even microbial infections (91–94). The roles of EVs in homeostasis must be balanced since EVs secreted from cells can travel through the circulatory system to deliver information to neighboring cells or cells in different locations (95).

**Figure 2:**
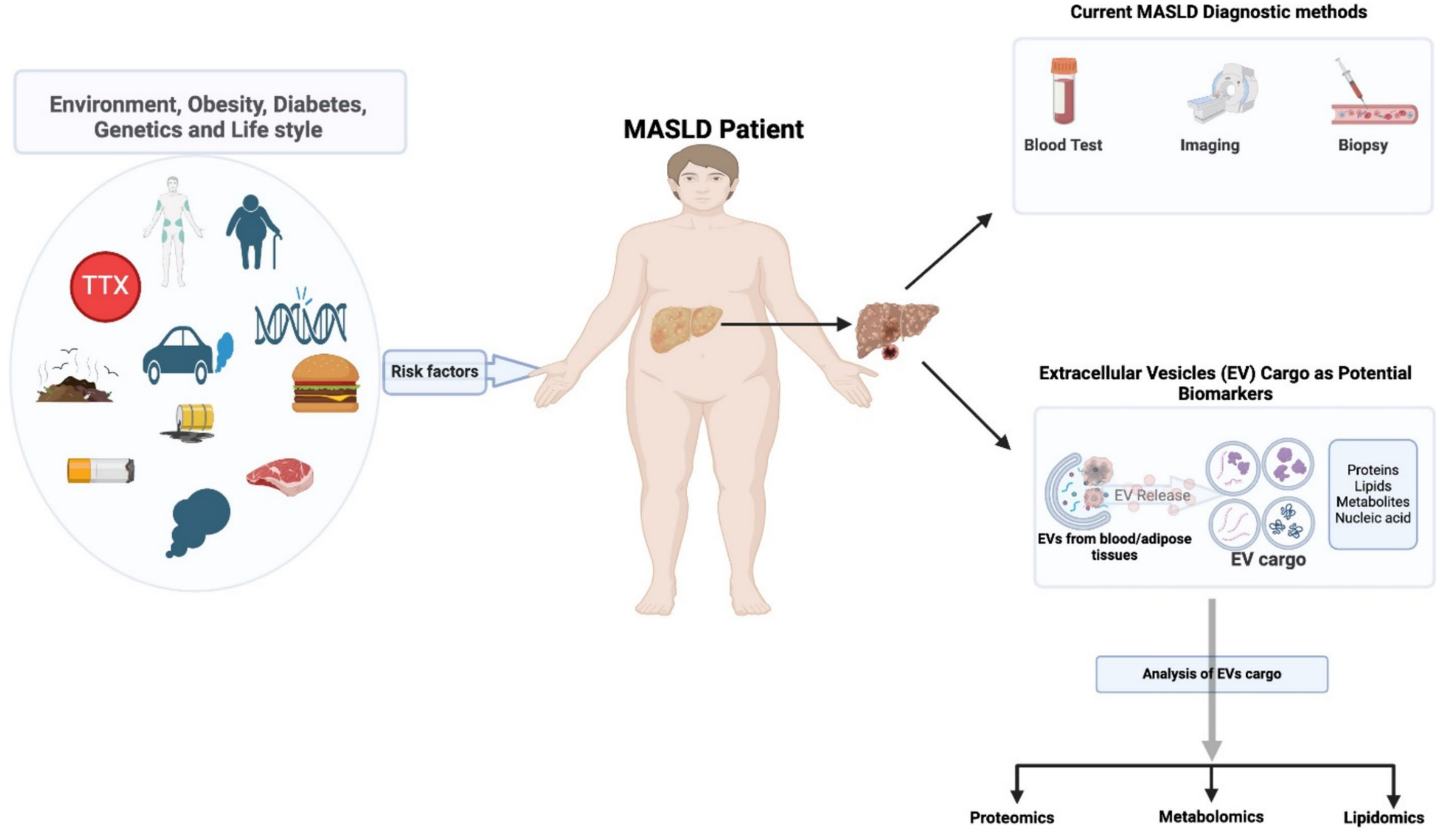
Extracellular vesicles from blood or adipose tissues of MASLD patients present the potential for biomarkers of MASLD.

EVs can also carry metabolites, RNAs, DNA, and miRNA cargo that may serve as MASLD biomarkers. Studies have shown that circulating miR-135a-3p in exosomes may be potential noninvasive biomarkers for diagnosing MASLD (96). This miRNA is a more sensitive and specific biological marker for MASLD than ALT (96). EV-omics—a subset of proteomics—may provide insight into cell–cell communication in hepatocytes during MASLD progression (Figure 2), presenting an excellent opportunity for noninvasive biomarkers that can be isolated from urine (97), saliva (98), CSF (99), and plasma (100).

### 5.2 Exposomics

Industrial and manufacturing sectors (gas, chemical plastics, energy, emissions, heavy machinery) have contributed to large-scale pollution, impacting water, air, and soil. While many initiatives and mitigation strategies have helped reduce pollution, more research is needed to identify the various environmental and chemical toxicants that impact human health. An environmental toxicant can be viewed as any toxic agent or substance produced by humans or introduced into the environment by human activities. Toxicants come in diverse shapes and sizes and may emanate from natural and anthropogenic sources. Impact on the physical environment includes thermal/climatic stress, altitude (hypoxia), natural disasters, radiation, pollution, and metal exposure. Particulate matter, polyaromatic hydrocarbons, chemical solvents, mycotoxins, phthalates, lead, organophosphates mercury, perfluorocarbons, polychlorinated biphenyls, cadmium, and arsenic are the most studied toxicants due to health concerns and disease associations.

Cells are subject to chemical, molecular, and physical stresses that can become toxic to metabolic processes, alter macromolecular interactions and signaling, affect pH, and more. These external stresses can become internal molecular stresses, producing reactive metabolic byproducts such as reactive oxidative species (ROS) and uninitiated cell death pathways. Cells and tissues’ intrinsic and adaptative ability to modulate these molecular processes in response to stresses is essential to maintaining homeostasis (101). Cell hemostasis regulation relies on the heat shock response, unfolded protein response, oxidative stress response, and DNA damage response. While these response pathways have been widely studied, emerging evidence on extracellular vesicles as a mode of disease transmission has garnered attention.

Human environmental exposures, often called the “exposome,” represent a broad spectrum of external and internal factors, including chemical, physical, biological, and social elements that can influence human health outcomes. These environmental factors can enter the body through various routes, such as inhalation, ingestion, and dermal absorption, exerting detrimental effects on liver health. The development of fatty liver disease has been linked to exposure to environmental contaminants, including air pollutants, heavy metals, pesticides, endocrine-disrupting agents, and environmental toxins (102, 103). These environmental exposures can cause inflammation, oxidative stress, and abnormal lipid metabolism in the liver, leading to hepatocyte fat buildup (104). The risk of fatty liver disease is further increased by lifestyle factors influenced by environmental factors, such as diet, physical activity, and socioeconomic status.

The exposome emphasizes the importance of considering external and internal environmental exposures and their interactions with genetic and epigenetic factors in shaping individual susceptibility to fatty liver disease. Understanding the complex interactions between environmental exposures and liver function is essential to prevent and treat MASLD effectively (104, 105). Particulate matter air pollution (PM2.5), derived mainly from fossil fuel combustion, is associated with MASLD. A recent cross-sectional study conducted on hospitalized patients in the United States Nationwide Inpatient Sample (NIS) database found a link between ambient PM2.5 exposure and an increased risk of MASLD (106), emphasizing the need for further investigation to examine the impact of eleven past long-term PM2.5 exposure events on incident instances of MASLD.

Exposure to heavy metals such as mercury (107), lead (108), arsenic (109), and iron (110) is associated with a higher incidence of fatty liver disease and liver dysfunction, which has been implicated in the onset and progression of MASLD in various communities. Endocrine disruptors (such as organochlorine pesticides that alter lipid metabolism and cause oxidative stress in the liver) are linked to a higher risk of fatty liver disease and liver dysfunction (111). Genetic variants involved in detoxification pathways and lipid metabolism may influence a person’s vulnerability to environmental toxins and ability to metabolize and eliminate environmental exposures (112). In environmental exposures, lifestyle factors, including diet, exercise, and alcohol intake, can alter the risk of fatty liver disease. Socioeconomic differences worsen the impacts of environmental pollutants since those from underprivileged backgrounds are more likely to be exposed to pollution at higher levels and to be more vulnerable to its harmful effects on liver health. To reduce the incidence of fatty liver disease and improve liver health across various demographics, targeted therapies and public health initiatives must consider the intricate interactions between host variables and environmental exposures

### 5.3 Single cell proteomics

In sophisticated biological systems such as the liver, characterized by its complex lobular architecture and numerous cell types, each type is assumed to have a unique function, lineage, and molecular profile, and assays of average cell populations adequately represent the fundamental biological processes within individual cells. Genetically identical cells, however, can differ significantly in function and composition, offering novel insights into hepatocellular dynamics. These discrepancies can profoundly affect the cell population’s functionality, contributing to MASLD progression. Mass spectrometry (MS)-based single-cell proteomics (SCP) provides in-depth revelations into cellular heterogeneity and has made remarkable advancements in recent decades. Initially focused on larger cells, recent improvements in MS experimental workflow, proof-of-concept studies, and sample preparation techniques enable the analysis of various cell types (113, 114). Modern SCP techniques can quantify approximately 1,000–1,500 proteins per single cell and up to 2,500 proteins across multiple cells (115). SCP could be highly beneficial in studying MASLD by enabling detailed mapping of protein expression in individual liver cells, thus allowing scientists to gain a deeper understanding of subpopulations of liver cells and identify dysregulated proteins and pathways that contribute to MASLD progression. SCP provides insights into lipid metabolism at the single-cell level. Abnormal lipid biosynthesis and metabolism are central to MASLD progression, and SCP can offer precise characterization of lipid isomers, including those differentiated by C=C bond and sn-position isomerism (115–117). By profiling lipid heterogeneity in liver cells, SCP could identify metabolic dysfunctions linked to MASLD, potentially revealing new biomarkers or therapeutic targets.

Single-cell proteomics presents considerable challenges due to the limited protein quantities and wide dynamic range of protein abundances within individual cells. Unlike nucleic acids, proteins cannot be amplified, necessitating highly sensitive analytical methods. Moreover, the technical demands of single-cell sampling and manipulation are exacerbated by small sample volumes and complex chemical environments, requiring precise extraction of analytes to prevent loss, dilution, or alterations to the cell’s native chemical profile (115). Currently, two key approaches to sample preparation—label-free and multiplexed methods—are undergoing active refinement (118). While challenges persist, such as the limited proteome depth achievable in each cell and the capacity to analyze only a few hundred cells per day, the potential of SCP remains immense (119). As these techniques evolve, SCP is poised to play a transformative role in shaping personalized medicine, advancing diagnostics, and driving therapeutic innovations (114, 118, 119). Label-free proteomics eliminates the need for chemical derivatization, a process that modifies proteins for detection, which can be complex and costly. Instead, it avoids using isotope-labeled reagents, making it more resource-efficient. In contrast, multiplexed single-cell proteomics employs tandem mass tags (TMTs) to label and analyze multiple samples in parallel, increasing throughput.

Another challenge to SCP, as in other omics areas, is standardizing the workflow and data processing to enable reproducible data (120, 121). Grégoire et al. (122) published a chapter detailing the R/Bioconductor package, scp, which provides a consistent framework for SCP data analysis using QFeatures and single-cell experiment structures. The approach includes a detailed protocol covering quality control, data aggregation, normalization, and batch correction, validated with controlled data sets, and fully outlines how to use the SCP package effectively. In a diet-induced MASH mouse model—known for mimicking the key features of human MASH, including steatosis, inflammation, and fibrosis—Ægidius et al. (123) integrated bulk RNA-seq, quantitative proteomics, and single-cell RNA-seq (scRNA-seq). The researchers developed a cell-type-specific map of liver pathology. They highlighted a disconnect between mRNA and protein levels in many cases, underscoring the importance of a multi-omics approach to fully capture the complexity of MASH. This liver single-cell atlas data is the closest clinical manifestation to human MASH. Due to liver inaccessibility, liver SCP studies are minimal. Weinberg et al. (124) stated, “Actual single-cell proteomics of human livers have not been done yet,” but stem cell and organoid technologies coupled with single-cell proteomics may help change that.

### 5.4 Proteogenomics

Proteogenomics is a multi-omics process that employs next-generation sequencing and mass spectrometry-based proteomics to integrate genomic, transcriptomic, and proteomic data to uncover novel proteins, improve disease processes, and identify potential biomarkers or therapeutic targets (125–127). This methodology bridges the gap between genotypic information and phenotypic protein expression, which enhances our ability to interpret variations in the genome by identifying how these changes manifest at the protein level, thus providing a more comprehensive view of cellular function and disease mechanisms.

Depending on the focus of the study and the available data, workflows are modifiable when integrating proteomics with transcriptomics. Generally, all workflows will normalize proteomic data and then combine the quantitative proteomics data with the quantitative transcriptomic data. Then, various outputs, like differential expression comparisons, network analysis, and functional annotations, allow researchers to gain a clearer picture of how proteins are expressed and how genetic variations influence disease (127, 128), detail the use of genomic data in proteogenomic biomarker discovery via several steps: Initially, the genomic sequencing data is aligned with a reference transcriptome to produce BAM files. Variants identified from these aligned reads are recorded in Variant Call Format (VCF) files and then translated into protein sequences FASTA files. Then, mass spectrometry techniques sequence proteins, employing data-dependent or independent acquisition methods. The resulting mass spectrometry data is matched against a custom-generated protein library based on genomic data. Potential biomarkers identified through this procedure are validated with targeted proteomics or antibody-based assays in extensive cohort studies. Proteogenomics requires sophisticated software tools for data integration, validation, and analysis because of the considerably sizeable datasets generated from DNA, RNA, and protein sequencing. This complexity presents a substantial challenge to the methodology. Conversely, this approach is precious in complex conditions such as MASLD, where genetic and molecular alterations play a critical role in disease progression and may lead to hepatocellular carcinoma (HCC) (129). The ability to identify specific protein variants in patients with MASLD offers essential insights into how these variants contribute to the disease’s advancement.

Research by Ægidius et al. (123) allows us to see how impactful proteogenomics is in their discovery of discrepancies between gene expression and protein levels for key molecules like Rbp4 and Erlin1, which are involved in lipid metabolism and inflammation. This underscores the significance of proteomic data in capturing post-transcriptional regulation, protein turnover, and other factors that influence protein abundance, which are critical for understanding the disease. Furthermore, this study by Ægidius et al. (123) is an excellent example of how the combination of proteogenomics technology enables researchers to capture the multifactorial complexity of the stages of MASLD and identify specific pathways involved in MASH pathogenesis, including those related to lipid metabolism, inflammation, mitochondrial dysfunction, and extracellular matrix production. In addition, Peiseler et al. (130) also validates how recent advances in technologies like proteogenomics have enhanced the understanding of the role immune cells, such as macrophages, T cells, and dendritic cells, play in MASLD. Their review identifies key immune cells contributing to different stages of MASLD progression by interacting with damaged hepatocytes, promoting fibrosis, and even influencing cancer development in MASH-associated HCC (130).

## 6 Future challenges

We must acknowledge the ethical implications of omics research. The Common Rule focuses on the role of respect, justice, and beneficence for omics research and outlines the need for informed consent, data sharing, trust, equal benefit, equal access, societal variables, privacy, data security, and participant feedback (131). Safeguards for ethical practices include Institutional Internal Review Boards, which oversee the documentation of any study and have in place informed consent from all participants to ensure they fully understand the study and that their participation is voluntary. Additionally, all biological samples obtained must be de-identified before any epidemiological, biochemical, or molecular analyses. Individual institution Offices of Sponsored Research (OSP) and federal funding agencies require data security, data sharing, and confidentiality agreements policies to ensure the safety of all participants’ personal information is secure (132).

Another challenge has been reproducibility in protein biomarker discovery, which can significantly impact the validity and reliability of findings. These issues arise from various factors, from technical variability, such as sample preparation and handling, to instrumentation differences across laboratories. Biological variability also creates issues with finding similar candidate biomarkers across large sample sizes, creating methodological issues and perturbations when sample sizes are statistically inadequate. Bias may arise from the interpretation of the resultant data, making it challenging to evaluate the actual reproducibility of biomarker discoveries, and reproducible biomarker discovery requires proper statistical validation. Large cohorts/population proteomics are challenging as samples can exceed 1,000 or more. Due to the decline of instrument performance sensitivity, proteomic data will contain missing peptide identifications or peak area values when obtained by data acquisition and processing pipeline methods. Instrument system performance of “housing keeping” peptides present in plasma or cell mixtures and the number of accurately quantified peptides can be used as a readout to observe peptides that fall below the detection limit to monitor sensitivity. Other challenges imposed by technical noise, inaccurate peak-picking algorithms, and incorrect computation of false discovery rates can also impact technical variation and will affect data reproducibility. Quality control experiments using standards can monitor fluctuations in signal-to-noise. As such, statistical normalization can be performed to remove technical variation for individual instruments. The coefficient of variation (CV) is a statistical tool used to assess the variability of data in proteomics and used to evaluate the performance of a LC/MS method or computational software used for protein/peptide quantitation. By measuring the standard deviation to the mean, it measures how close the multiple measurements from LC/MS experiments from different samples are to each other.

Proteomics has evolved and been used over the years in a variety of approaches to monitor or understand quantitative changes in protein expression that may occur due to disease conditions, the body’s response to exposure to drugs or toxins, gene-by-environment changes and the impacts of these changes in understanding the pathophysiology of different disease conditions and their relationship to the environment (133, 134). The advancement in quantitative and qualitative mass spectrometry-based proteomics has tremendously contributed to understanding multiple cellular phenotypes. Even though mass spectrometry-based proteomics holds great potential for facilitating the identification of protein biomarkers, in the last 10 years, few novel biomarkers have been brought into clinical use. Moreover, the utility of data complexity and interpretation represents a limitation. Finding a limited number of viable candidates from thousands of proteins identified by untargeted MS proteomics for further validation and verification using targeted assays is one of the rate-limiting phases in discovering protein biomarkers. Another issue has been reproducibility in protein biomarker discovery, which can significantly impact the validity and reliability of findings. These issues arise from various factors, from technical variability, such as sample preparation and handling, to instrumentation differences across laboratories. Biological variability also creates issues with finding similar candidate biomarkers across large sample sizes, creating methodological issues and perturbations when sample sizes are statistically inadequate. Bias may arise from the interpretation of the resultant data, including variations in ethnic background, exposure to social determinants of health (135, 136), and health-related social needs, making it challenging to evaluate the actual reproducibility of biomarker discoveries and reproducible biomarker discovery requires proper statistical validation.

It is of utmost importance to utilize and advance technology research to better detect and differentiate the various stages of MASLD with reliable, affordable, minimally intensive, risk-free screening applications to diagnose and predict the risk of MASLD in daily clinical routine. To date, there are currently minimal treatment options for MASLD. Research on multi-omics is on track to identify targeted precision medicine technology for determining etiological risk, early identification, stratification, treatment, and research on MASLD.

## 7 Conclusion

Metabolic Dysfunction-Associated Steatotic Liver Disease (MASLD) remains a significant health concern, paralleling the global obesity epidemic. Integrating modern diagnostic imaging techniques and innovative proteomic methods is crucial for advancing our understanding and management of MASLD’s complex pathophysiology. Technology not only facilitates the discovery of novel biomarkers but also enables faster, less invasive, and more accurate diagnosis, disease severity assessment, and treatment efficacy evaluation. The application of proteomics, particularly mass spectrometry-based approaches, is instrumental in identifying circulating protein signatures associated with MASLD progression. This has opened avenues for developing noninvasive diagnostic tools like blood-based biomarker panels. Proteomics has the potential to uncover additional causes of MASLD by elucidating gene-by-environment interactions and identifying key proteins involved in disease progression. Future research must continue to leverage these technologies to enhance early detection, personalized treatment strategies, and monitor therapeutic responses in real-time. By emphasizing the role of modern diagnostic imaging and proteomics, we can accelerate the development of targeted therapies and improve clinical outcomes for MASLD patients.

Additionally, proteomics has expanded into subspecialties such as EV-omics, single cell, and proteogenomics, each with the potential to identify more novel biomarkers. Identifying and quantifying nucleic acids, proteins, and metabolites will continue to contribute valuable insights in exposomics, particularly for environmental toxicants and drug toxicity screening. Therefore, performing deeper level analysis of molecular networks and integrating statistical gene–environment interactions will be crucial for finding effective treatments and understanding the causative drivers of MASLD.

## References

[1] Juanola O Martínez-López S Francés R Gómez-Hurtado I. Non-alcoholic fatty liver disease: metabolic, genetic, epigenetic and environmental risk factors. Int J Environ Res Public Health. (2021) 18:227. doi: 10.3390/ijerph18105227, PMID: 34069012 PMC8155932

[2] Manusov EG Diego VP Smith J Garza JR Lowdermilk J Blangero J. UniMóvil: a Mobile health clinic providing primary care to the Colonias of the Rio Grande Valley, South Texas. Front Public Health. (2019) 7:215. doi: 10.3389/fpubh.2019.00215, PMID: 31497586 PMC6712363

[3] Targher G Corey KE Byrne CD. NAFLD, and cardiovascular and cardiac diseases: factors influencing risk, prediction and treatment. Diabetes Metab. (2021) 47:101215. doi: 10.1016/j.diabet.2020.101215, PMID: 33296704

[4] Chalasani N Younossi Z Lavine JE Charlton M Cusi K Rinella M. The diagnosis and management of nonalcoholic fatty liver disease: practice guidance from the American Association for the Study of Liver Diseases. Hepatology. (2018) 67:328–57. doi: 10.1002/hep.29367, PMID: 28714183

[5] Bessone F Razori MV Roma MG. Molecular pathways of nonalcoholic fatty liver disease development and progression. Cell Mol Life Sci. (2019) 76:99–128. doi: 10.1007/s00018-018-2947-0, PMID: 30343320 PMC11105781

[6] Younossi ZM Blissett D Blissett R Henry L Stepanova M Younossi Y. The economic and clinical burden of nonalcoholic fatty liver disease in the United States and Europe. Hepatology. (2016) 64:1577–86. doi: 10.1002/hep.28785, PMID: 27543837

[7] Chan KE Koh TJL Tang ASP Quek J Yong JN Tay P. Global prevalence and clinical characteristics of metabolic-associated fatty liver disease: a Meta-analysis and systematic review of 10 739 607 individuals. J Clin Endocrinol Metab. (2022) 107:2691–700. doi: 10.1210/clinem/dgac321, PMID: 35587339

[8] Teng ML Ng CH Huang DQ Chan KE Tan DJH Lim WH. Global incidence and prevalence of nonalcoholic fatty liver disease. Clin Mol Hepatol. (2023) 29:S32–42. doi: 10.3350/cmh.2022.0365, PMID: 36517002 PMC10029957

[9] Olvera RL Williamson DE Fisher-Hoch SP Vatcheva KP McCormick JB. Depression, obesity, and metabolic syndrome: prevalence and risks of comorbidity in a population-based representative sample of Mexican Americans. J Clin Psychiatry. (2015) 76:e1300–5. doi: 10.4088/JCP.14m09118, PMID: 26528653 PMC5836315

[10] Niu L Geyer PE Wewer Albrechtsen NJ Gluud LL Santos A Doll S. Plasma proteome profiling discovers novel proteins associated with non-alcoholic fatty liver disease. Mol Syst Biol. (2019) 15:e8793. doi: 10.15252/msb.20188793, PMID: 30824564 PMC6396370

[11] Kawano Y Cohen DE. Mechanisms of hepatic triglyceride accumulation in non-alcoholic fatty liver disease. J Gastroenterol. (2013) 48:434–41. doi: 10.1007/s00535-013-0758-5, PMID: 23397118 PMC3633701

[12] Ciardullo S. Commentary on Kim et al. silent threats: how NAFLD and hypertension team up in young adults, and the role of sex. Nutr Metab Cardiovasc Dis. (2023) 33:1617–8. doi: 10.1016/j.numecd.2023.05.021, PMID: 37336717

[13] Makri E Goulas A Polyzos SA. Epidemiology, pathogenesis, diagnosis and emerging treatment of nonalcoholic fatty liver disease. Arch Med Res. (2021) 52:25–37. doi: 10.1016/j.arcmed.2020.11.010, PMID: 33334622

[14] Zhang J Zhou J He Z Li H. Bacteroides and NAFLD: pathophysiology and therapy. Front Microbiol. (2024) 15:1288856. doi: 10.3389/fmicb.2024.1288856, PMID: 38572244 PMC10988783

[15] Valenzuela-Vallejo L Mantzoros CS. Time to transition from a negative nomenclature describing what NAFLD is not, to a novel, pathophysiology-based, umbrella classification of fatty liver disease (FLD). Metabolism. (2022) 134:155246. doi: 10.1016/j.metabol.2022.155246, PMID: 35780909

[16] Fracanzani AL Valenti L Bugianesi E Andreoletti M Colli A Vanni E. Risk of severe liver disease in nonalcoholic fatty liver disease with normal aminotransferase levels: a role for insulin resistance and diabetes. Hepatology. (2008) 48:792–8. doi: 10.1002/hep.22429, PMID: 18752331

[17] Reinson T Buchanan RM Byrne CD. Noninvasive serum biomarkers for liver fibrosis in NAFLD: current and future. Clin Mol Hepatol. (2023) 29:S157–s170. doi: 10.3350/cmh.2022.0348, PMID: 36417894 PMC10029954

[18] Noureddin M Truong E Gornbein JA Saouaf R Guindi M Todo T. MRI-based (MAST) score accurately identifies patients with NASH and significant fibrosis. J Hepatol. (2022) 76:781–7. doi: 10.1016/j.jhep.2021.11.012, PMID: 34798176

[19] Woreta TA van Natta ML Lazo M Krishnan A Neuschwander-Tetri BA Loomba R. Validation of the accuracy of the FAST™ score for detecting patients with at-risk nonalcoholic steatohepatitis (NASH) in a north American cohort and comparison to other non-invasive algorithms. PLoS One. (2022) 17:e0266859. doi: 10.1371/journal.pone.0266859, PMID: 35427375 PMC9012361

[20] Cassinotto C Boursier J de Lédinghen V Lebigot J Lapuyade B Cales P. Liver stiffness in nonalcoholic fatty liver disease: a comparison of supersonic shear imaging, FibroScan, and ARFI with liver biopsy. Hepatology. (2016) 63:1817–27. doi: 10.1002/hep.28394, PMID: 26659452

[21] Guo L Zheng L Hu L Zhou H Yu L Liang W. Transient Elastography (FibroScan) performs better than non-invasive markers in assessing liver fibrosis and cirrhosis in autoimmune hepatitis patients. Med Sci Monit. (2017) 23:5106–12. doi: 10.12659/MSM.907300, PMID: 29073121 PMC5669534

[22] Patel K Wilder J. Fibroscan. Clin Liver Dis. (2014) 4:97–101. doi: 10.1002/cld.407, PMID: 30992931 PMC6448744

[23] Xu Y Liu Y Cao Z Wang L Li Z Sheng Z. Comparison of FibroTouch and FibroScan for staging fibrosis in chronic liver disease: single-center prospective study. Dig Liver Dis. (2019) 51:1323–9. doi: 10.1016/j.dld.2019.02.009, PMID: 30928419

[24] Machado MV Cortez-Pinto H. Non-invasive diagnosis of non-alcoholic fatty liver disease: A critical appraisal. J Hepatol. (2013) 58:1007–19. doi: 10.1016/j.jhep.2012.11.021, PMID: 23183525

[25] Kanwal F Shubrook JH Adams LA Pfotenhauer K Wai-Sun Wong V Wright E. Clinical care pathway for the risk stratification and Management of Patients with Nonalcoholic Fatty Liver Disease. Gastroenterology. (2021) 161:1657–69. doi: 10.1053/j.gastro.2021.07.049, PMID: 34602251 PMC8819923

[26] Wei S Wang L Evans PC Xu S. NAFLD and NASH: etiology, targets and emerging therapies. Drug Discov Today. (2024) 29:103910. doi: 10.1016/j.drudis.2024.103910, PMID: 38301798

[27] la X Zhang Z Liang J Li H Pang Y He X. Isolation and purification of flavonoids from quinoa whole grain and its inhibitory effect on lipid accumulation in nonalcoholic fatty liver disease by inhibiting the expression of CD36 and FASN. J Sci Food Agric. (2025) 105:1330–42. doi: 10.1002/jsfa.13923, PMID: 39305086

[28] Chen S Lu H Yin G Zhang X Meng D Yu W. Hesperitin prevents non-alcoholic steatohepatitis by modulating mitochondrial dynamics and mitophagy via the AMPKα-Drp1/PINK1-Parkin signaling pathway. Biochim Biophys Acta Mol Cell Biol Lipids. (2025) 1870:159570. doi: 10.1016/j.bbalip.2024.159570, PMID: 39454819

[29] Friedman SL Neuschwander-Tetri BA Rinella M Sanyal AJ. Mechanisms of NAFLD development and therapeutic strategies. Nat Med. (2018) 24:908–22. doi: 10.1038/s41591-018-0104-9, PMID: 29967350 PMC6553468

[30] Dudeja P Pal T Sharma A. The novel approach for non-invasive diagnostic biomarkers from an early stage of NAFLD to advanced fibrosis. Egypt Liver J. (2023) 13:51. doi: 10.1186/s43066-023-00287-3

[31] Masoodi M Gastaldelli A Hyötyläinen T Arretxe E Alonso C Gaggini M. Metabolomics and lipidomics in NAFLD: biomarkers and non-invasive diagnostic tests. Nat Rev Gastroenterol Hepatol. (2021) 18:835–56. doi: 10.1038/s41575-021-00502-9, PMID: 34508238

[32] Harrison SA Ratziu V Magnanensi J Hajji Y Deledicque S Majd Z. NIS2+™, an optimisation of the blood-based biomarker NIS4® technology for the detection of at-risk NASH: a prospective derivation and validation study. J Hepatol. (2023) 79:758–67. doi: 10.1016/j.jhep.2023.04.031, PMID: 37224923

[33] Ratziu V Harrison SA Hajji Y Magnanensi J Petit S Majd Z. NIS2+(TM) as a screening tool to optimize patient selection in metabolic dysfunction-associated steatohepatitis clinical trials. J Hepatol. (2024) 80:209–19. doi: 10.1016/j.jhep.2023.10.038, PMID: 38061448

[34] Castera L Friedrich-Rust M Loomba R. Noninvasive assessment of liver disease in patients with nonalcoholic fatty liver disease. Gastroenterology. (2019) 156:1264–1281.e4. doi: 10.1053/j.gastro.2018.12.036, PMID: 30660725 PMC7505052

[35] Wattacheril JJ Abdelmalek MF Lim JK Sanyal AJ. AGA clinical practice update on the role of noninvasive biomarkers in the evaluation and Management of Nonalcoholic Fatty Liver Disease: expert review. Gastroenterology. (2023) 165:1080–8. doi: 10.1053/j.gastro.2023.06.013, PMID: 37542503

[36] Govaere O Hasoon M Alexander L Cockell S Tiniakos D Ekstedt M. A proteo-transcriptomic map of non-alcoholic fatty liver disease signatures. Nat Metab. (2023) 5:572–8. doi: 10.1038/s42255-023-00775-1, PMID: 37037945 PMC10132975

[37] Ou FS Michiels S Shyr Y Adjei AA Oberg AL. Biomarker discovery and validation: statistical considerations. J Thorac Oncol. (2021) 16:537–45. doi: 10.1016/j.jtho.2021.01.1616, PMID: 33545385 PMC8012218

[38] das S Dey M Devireddy R Gartia M. Biomarkers in Cancer detection, diagnosis, and prognosis. Sensors. (2023) 24:37. doi: 10.3390/s24010037, PMID: 38202898 PMC10780704

[39] al-Tashi Q Saad M Muneer A Qureshi R Mirjalili S Sheshadri A. Machine learning models for the identification of prognostic and predictive Cancer biomarkers: a systematic review. Int J Mol Sci. (2023) 24:781. doi: 10.3390/ijms24097781, PMID: 37175487 PMC10178491

[40] Pal M Muinao T Boruah HPD Mahindroo N. Current advances in prognostic and diagnostic biomarkers for solid cancers: detection techniques and future challenges. Biomed Pharmacother. (2022) 146:112488. doi: 10.1016/j.biopha.2021.112488, PMID: 34894516

[41] Pozzi L., *Types of biomarkers*, in *atlas antibodies*. (2024).

[42] Shin D Kim Y Park J Kim Y. High-throughput proteomics-guided biomarker discovery of hepatocellular carcinoma. Biom J. (2024) 48:100752. doi: 10.1016/j.bj.2024.100752 PMC11743302 38901798

[43] Wiśniewski JR Duś K Mann M. Proteomic workflow for analysis of archival formalin-fixed and paraffin-embedded clinical samples to a depth of 10 000 proteins. Proteomics Clin Appl. (2013) 7:225–33. doi: 10.1002/prca.201200046, PMID: 23090905

[44] Abdelhameed F Kite C Lagojda L Dallaway A Chatha KK Chaggar SS. Non-invasive scores and serum biomarkers for fatty liver in the era of metabolic dysfunction-associated Steatotic liver disease (MASLD): a comprehensive review from NAFLD to MAFLD and MASLD. Curr Obes Rep. (2024) 13:510–31. doi: 10.1007/s13679-024-00574-z, PMID: 38809396 PMC11306269

[45] Xing X Cai L Ouyang J Wang F Li Z Liu M. Proteomics-driven noninvasive screening of circulating serum protein panels for the early diagnosis of hepatocellular carcinoma. Nat Commun. (2023) 14:8392. doi: 10.1038/s41467-023-44255-2, PMID: 38110372 PMC10728065

[46] Thomas SN French D Jannetto PJ Rappold BA Clarke WA. Liquid chromatography-tandem mass spectrometry for clinical diagnostics. Nat Rev Methods Primers. (2022) 2:96. doi: 10.1038/s43586-022-00175-x, PMID: 36532107 PMC9735147

[47] Marquioni V Nunes FMF Novo-Mansur MTM. Protein identification by database searching of mass spectrometry data in the teaching of proteomics. J Chem Educ. (2021) 98:812–23. doi: 10.1021/acs.jchemed.0c00853

[48] Bowler-Barnett EH Fan J Luo J Magrane M Martin MJ Orchard S. UniProt and mass spectrometry-based proteomics-a 2-way working relationship. Mol Cell Proteomics. (2023) 22:100591. doi: 10.1016/j.mcpro.2023.100591, PMID: 37301379 PMC10404557

[49] Khatri P Sirota M Butte AJ. Ten years of pathway analysis: current approaches and outstanding challenges. PLoS Comput Biol. (2012) 8:e1002375. doi: 10.1371/journal.pcbi.1002375, PMID: 22383865 PMC3285573

[50] Lin Z Wei L Cai W Zhu Y Tucholski T Mitchell SD. Simultaneous quantification of protein expression and modifications by top-down targeted proteomics: a case of the Sarcomeric subproteome. Mol Cell Proteomics. (2019) 18:594–605. doi: 10.1074/mcp.TIR118.001086, PMID: 30591534 PMC6398208

[51] Angelini G Panunzi S Castagneto-Gissey L Pellicanò F de Gaetano A Pompili M. Accurate liquid biopsy for the diagnosis of non-alcoholic steatohepatitis and liver fibrosis. Gut. (2023) 72:392–403. doi: 10.1136/gutjnl-2022-327498, PMID: 35820779 PMC9872242

[52] Gobena S Admassu B Kinde MZ Gessese AT. Proteomics and its current application in biomedical area: concise review. Sci World J. (2024) 2024:1–13. doi: 10.1155/2024/4454744, PMID: 38404932 PMC10894052

[53] Strimbu K Tavel JA. What are biomarkers? Curr Opin HIV AIDS. (2010) 5:463–6. doi: 10.1097/COH.0b013e32833ed177, PMID: 20978388 PMC3078627

[54] Boccardi M Dodich A Albanese E Gayet-Ageron A Festari C Ashton NJ. The strategic biomarker roadmap for the validation of Alzheimer's diagnostic biomarkers: methodological update. Eur J Nucl Med Mol Imaging. (2021) 48:2070–85. doi: 10.1007/s00259-020-05120-2, PMID: 33688996 PMC8175304

[55] Pérez Compte D Etourneau L Hesse AM Kraut A Barthelon J Sturm N. Plasma ALS and gal-3BP differentiate early from advanced liver fibrosis in MASLD patients. Biomark Res. (2024) 12:44. doi: 10.1186/s40364-024-00583-z, PMID: 38679739 PMC11057169

[56] Guzmán C Bautista-Ubaldo MG Campos-Espinosa A Romero-Bello II Santana-Vargas ÁD Gutierrez-Reyes G. Insulin-like growth factor binding proteins and cellular senescence are involved in the progression of non-alcoholic fatty liver disease and fibrosis in a mouse model. Medicina. (2024) 60:429. doi: 10.3390/medicina60030429 38541155 PMC10972469

[57] Ichikawa T Nakao K Hamasaki K Furukawa R Tsuruta S Ueda Y. Role of growth hormone, insulin-like growth factor 1 and insulin-like growth factor-binding protein 3 in development of non-alcoholic fatty liver disease. Hepatol Int. (2007) 1:287–94. doi: 10.1007/s12072-007-9007-4, PMID: 19669352 PMC2716823

[58] Corey KE Pitts R Lai M Loureiro J Masia R Osganian SA. ADAMTSL2 protein and a soluble biomarker signature identify at-risk non-alcoholic steatohepatitis and fibrosis in adults with NAFLD. J Hepatol. (2022) 76:25–33. doi: 10.1016/j.jhep.2021.09.026, PMID: 34600973 PMC8688231

[59] Cao Y du Y Jia W Ding J Yuan J Zhang H. Identification of biomarkers for the diagnosis of chronic kidney disease (CKD) with non-alcoholic fatty liver disease (NAFLD) by bioinformatics analysis and machine learning. Front Endocrinol. (2023) 14:1125829. doi: 10.3389/fendo.2023.1125829, PMID: 36923221 PMC10009268

[60] di S Scamporrino A Filippello A di A Scicali R Malaguarnera R. Clinical and molecular biomarkers for diagnosis and staging of NAFLD. Int J Mol Sci. (2021) 22:905. doi: 10.3390/ijms222111905, PMID: 34769333 PMC8585051

[61] Nassir F. NAFLD: mechanisms, treatments, and biomarkers. Biomol Ther. (2022) 12:824. doi: 10.3390/biom12060824, PMID: 35740949 PMC9221336

[62] Wong VW Adams LA de Lédinghen V Wong GL Sookoian S. Noninvasive biomarkers in NAFLD and NASH - current progress and future promise. Nat Rev Gastroenterol Hepatol. (2018) 15:461–78. doi: 10.1038/s41575-018-0014-9, PMID: 29844588

[63] Lim JW Dillon J Miller M. Proteomic and genomic studies of non-alcoholic fatty liver disease--clues in the pathogenesis. World J Gastroenterol. (2014) 20:8325–40. doi: 10.3748/wjg.v20.i26.8325, PMID: 25024592 PMC4093687

[64] Jiang Y Zhuang X Zhang J Li M du S Tian J. Clinical characterization and proteomic profiling of lean nonalcoholic fatty liver disease. Front Endocrinol. (2023) 14:1171397. doi: 10.3389/fendo.2023.1171397, PMID: 38034020 PMC10687542

[65] Rodríguez-Suárez E Duce AM Caballería J Arrieta FM Fernández E Gómara C. Non-alcoholic fatty liver disease proteomics. Proteomics Clin Appl. (2010) 4:362–71. doi: 10.1002/prca.200900119, PMID: 21137056 PMC3040121

[66] Robinson AE Binek A Ramani K Sundararaman N Barbier-Torres L Murray B. Hyperphosphorylation of hepatic proteome characterizes nonalcoholic fatty liver disease in S-adenosylmethionine deficiency. iScience. (2023) 26:105987. doi: 10.1016/j.isci.2023.105987, PMID: 36756374 PMC9900401

[67] Altomare AA Aiello G Garcia JL Garrone G Zoanni B Carini M. Protein profiling of a cellular model of NAFLD by advanced bioanalytical approaches. Int J Mol Sci. (2022) 23:25. doi: 10.3390/ijms23169025, PMID: 36012291 PMC9408868

[68] Haukeland JW Damås JK Konopski Z Løberg EM Haaland T Goverud I. Systemic inflammation in nonalcoholic fatty liver disease is characterized by elevated levels of CCL2. J Hepatol. (2006) 44:1167–74. doi: 10.1016/j.jhep.2006.02.011, PMID: 16618517

[69] Coulon S Francque S Colle I Verrijken A Blomme B Heindryckx F. Evaluation of inflammatory and angiogenic factors in patients with non-alcoholic fatty liver disease. Cytokine. (2012) 59:442–9. doi: 10.1016/j.cyto.2012.05.001, PMID: 22658783

[70] Das SK Balakrishnan V. Role of cytokines in the pathogenesis of non-alcoholic fatty liver disease. Indian J Clin Biochem. (2011) 26:202–9. doi: 10.1007/s12291-011-0121-7, PMID: 22468051 PMC3107419

[71] Kar S Paglialunga S Jaycox SH Islam R Paredes AH. Assay validation and clinical performance of chronic inflammatory and chemokine biomarkers of NASH fibrosis. PLoS One. (2019) 14:e0217263. doi: 10.1371/journal.pone.0217263, PMID: 31291245 PMC6619600

[72] Yoshiji H Kuriyama S Miyamoto Y Thorgeirsson UP Gomez DE Kawata M. Tissue inhibitor of metalloproteinases-1 promotes liver fibrosis development in a transgenic mouse model. Hepatology. (2000) 32:1248–54. doi: 10.1053/jhep.2000.20521, PMID: 11093731

[73] Benyon RC Iredale JP Goddard S Winwood PJ Arthur MJ. Expression of tissue inhibitor of metalloproteinases 1 and 2 is increased in fibrotic human liver. Gastroenterology. (1996) 110:821–31. doi: 10.1053/gast.1996.v110.pm8608892, PMID: 8608892

[74] Busk TM Bendtsen F Nielsen HJ Jensen V Brünner N Møller S. TIMP-1 in patients with cirrhosis: relation to liver dysfunction, portal hypertension, and hemodynamic changes. Scand J Gastroenterol. (2014) 49:1103–10. doi: 10.3109/00365521.2014.934910, PMID: 25048331

[75] Hochreuter MY Dall M Treebak JT Barrès R. MicroRNAs in non-alcoholic fatty liver disease: Progress and perspectives. Mol Metab. (2022) 65:101581. doi: 10.1016/j.molmet.2022.101581, PMID: 36028120 PMC9464960

[76] Buniatian GH Schwinghammer U Tremmel R Cynis H Weiss TS Weiskirchen R. Consequences of amyloid-β deficiency for the liver. Adv Sci. (2024) 11:e2307734. doi: 10.1002/advs.202307734 PMC11095235 38430535

[77] Gross S Danielyan L Buechler C Kubitza M Klein K Schwab M. Hepatic amyloid Beta-42-metabolizing proteins in liver steatosis and metabolic dysfunction-associated Steatohepatitis. Int J Mol Sci. (2024) 25:768. doi: 10.3390/ijms25168768, PMID: 39201455 PMC11354580

[78] Guo Y Wang Q Chen S Xu C. Functions of amyloid precursor protein in metabolic diseases. Metabolism. (2021) 115:154454. doi: 10.1016/j.metabol.2020.154454, PMID: 33248065

[79] Ipsen DH Tveden-Nyborg P. Extracellular vesicles as drivers of non-alcoholic fatty liver disease: small particles with big impact. Biomedicines. (2021) 9:93. doi: 10.3390/biomedicines9010093, PMID: 33477873 PMC7832840

[80] Yang M Xu D Liu Y Guo X Li W Guo C. Combined serum biomarkers in non-invasive diagnosis of non-alcoholic Steatohepatitis. PLoS One. (2015) 10:e0131664. doi: 10.1371/journal.pone.0131664, PMID: 26121037 PMC4486729

[81] Mayo R Crespo J Martínez-Arranz I Banales JM Arias M Mincholé I. Metabolomic-based noninvasive serum test to diagnose nonalcoholic steatohepatitis: results from discovery and validation cohorts. Hepatol Commun. (2018) 2:807–20. doi: 10.1002/hep4.1188, PMID: 30027139 PMC6049064

[82] Zaborowski MP Balaj L Breakefield XO Lai CP. Extracellular vesicles: composition, biological relevance, and methods of study. Bioscience. (2015) 65:783–97. doi: 10.1093/biosci/biv084, PMID: 26955082 PMC4776721

[83] Raposo G Stoorvogel W. Extracellular vesicles: exosomes, microvesicles, and friends. J Cell Biol. (2013) 200:373–83. doi: 10.1083/jcb.201211138, PMID: 23420871 PMC3575529

[84] Kalluri R LeBleu VS. The biology, function, and biomedical applications of exosomes. Science. (2020) 367:aau6977. doi: 10.1126/science.aau6977, PMID: 32029601 PMC7717626

[85] Han C Yang J Sun J Qin G. Extracellular vesicles in cardiovascular disease: biological functions and therapeutic implications. Pharmacol Ther. (2022) 233:108025. doi: 10.1016/j.pharmthera.2021.108025, PMID: 34687770 PMC9018895

[86] Hill AF. Extracellular vesicles and neurodegenerative diseases. J Neurosci. (2019) 39:9269–73. doi: 10.1523/JNEUROSCI.0147-18.2019, PMID: 31748282 PMC6867808

[87] Takeuchi T. Pathogenic and protective roles of extracellular vesicles in neurodegenerative diseases. J Biochem. (2021) 169:181–6. doi: 10.1093/jb/mvaa131, PMID: 33196835

[88] Marar C Starich B Wirtz D. Extracellular vesicles in immunomodulation and tumor progression. Nat Immunol. (2021) 22:560–70. doi: 10.1038/s41590-021-00899-0, PMID: 33753940 PMC9389600

[89] El Andaloussi S Mäger I Breakefield XO Wood MJ. Extracellular vesicles: biology and emerging therapeutic opportunities. Nat Rev Drug Discov. (2013) 12:347–57. doi: 10.1038/nrd3978, PMID: 23584393

[90] Jóźwicka TM Erdmańska PM Stachowicz-Karpińska A Olkiewicz M Jóźwicki W. Exosomes-promising carriers for regulatory therapy in oncology. Cancers. (2024) 16:923. doi: 10.3390/cancers16050923, PMID: 38473285 PMC10931160

[91] de Oliveira HC Castelli RF Reis FCG Rizzo J Rodrigues ML. Pathogenic delivery: the biological roles of Cryptococcal extracellular vesicles. Pathogens. (2020) 9:754. doi: 10.3390/pathogens9090754, PMID: 32948010 PMC7557404

[92] Kim G Chen X Yang Y. Pathogenic extracellular vesicle (EV) signaling in amyotrophic lateral sclerosis (ALS). Neurotherapeutics. (2022) 19:1119–32. doi: 10.1007/s13311-022-01232-9, PMID: 35426061 PMC9587178

[93] Zhou X Liu S Lu Y Wan M Cheng J Liu J. MitoEVs: a new player in multiple disease pathology and treatment. J Extracell Vesicles. (2023) 12:e12320. doi: 10.1002/jev2.12320, PMID: 37002588 PMC10065981

[94] Tarashi S Zamani MS Omrani MD Fateh A Moshiri A Saedisomeolia A. Commensal and pathogenic bacterial-derived extracellular vesicles in host-bacterial and Interbacterial dialogues: two sides of the same coin. J Immunol Res. (2022) 2022:8092170. doi: 10.1155/2022/8092170 35224113 PMC8872691

[95] Carberry CK Keshava D Payton A Smith GJ Rager JE. Approaches to incorporate extracellular vesicles into exposure science, toxicology, and public health research. J Expo Sci Environ Epidemiol. (2022) 32:647–59. doi: 10.1038/s41370-022-00417-w, PMID: 35217808 PMC9402811

[96] Jiang H Qian Y Shen Z Liu Y He Y Gao R. Circulating microRNA-135a-3p in serum extracellular vesicles as a potential biological marker of non-alcoholic fatty liver disease. Mol Med Rep. (2021) 24:137. doi: 10.3892/mmr.2021.12137, PMID: 33955511 PMC8127071

[97] Zhu Q Li H Ao Z Xu H luo J Kaurich C. Lipidomic identification of urinary extracellular vesicles for non-alcoholic steatohepatitis diagnosis. J Nanobiotechnol. (2022) 20:349. doi: 10.1186/s12951-022-01540-4, PMID: 35897102 PMC9327366

[98] Schou AS Nielsen JE Askeland A Jørgensen MM. Extracellular vesicle-associated proteins as potential biomarkers. Adv Clin Chem. (2020) 99:1–48. doi: 10.1016/bs.acc.2020.02.011, PMID: 32951635

[99] Li Z Wang X Wang X Yi X Wong YK Wu J. Research progress on the role of extracellular vesicles in neurodegenerative diseases. Translational Neurodegeneration. (2023) 12:43. doi: 10.1186/s40035-023-00375-9, PMID: 37697342 PMC10494410

[100] Nguyen HQ Lee D Kim Y Bang G Cho K Lee YS. Label-free quantitative proteomic analysis of serum extracellular vesicles differentiating patients of alcoholic and nonalcoholic fatty liver diseases. J Proteome. (2021) 245:104278. doi: 10.1016/j.jprot.2021.104278, PMID: 34089894 PMC8277700

[101] Kültz D. Molecular and evolutionary basis of the cellular stress response. Annu Rev Physiol. (2005) 67:225–57. doi: 10.1146/annurev.physiol.67.040403.103635, PMID: 15709958

[102] Chen Y Wang Y Cui Z Liu W Liu B Zeng Q. Endocrine disrupting chemicals: a promoter of non-alcoholic fatty liver disease. Front Public Health. (2023) 11:1154837. doi: 10.3389/fpubh.2023.1154837, PMID: 37033031 PMC10075363

[103] Matthiessen C Glaubitz L Lucht S Kälsch J Luedde T Erbel R. Long-term exposure to air pollution and prevalent nonalcoholic fatty liver disease. Environ Epidemiol. (2023) 7:e268. doi: 10.1097/EE9.0000000000000268, PMID: 37840860 PMC10569764

[104] Barouki R Samson M Blanc EB Colombo M Zucman-Rossi J Lazaridis KN. The exposome and liver disease - how environmental factors affect liver health. J Hepatol. (2023) 79:492–505. doi: 10.1016/j.jhep.2023.02.034, PMID: 36889360 PMC10448911

[105] Ma N Yip R Lewis S Dinani A Wyatt C Crane M. Environmental exposures are important risk factors for advanced liver fibrosis in African American adults. JHEP Rep. (2023) 5:100696. doi: 10.1016/j.jhepr.2023.100696, PMID: 36937989 PMC10017423

[106] VoPham T Kim NJ Berry K Mendoza JA Kaufman JD Ioannou GN. PM(2.5) air pollution exposure and nonalcoholic fatty liver disease in the Nationwide inpatient sample. Environ Res. (2022) 213:113611. doi: 10.1016/j.envres.2022.113611, PMID: 35688225 PMC9378584

[107] Yang YJ Yang EJ Park K Oh S Kim T Hong YP. Association between blood mercury levels and non-alcoholic fatty liver disease in non-obese populations: the Korean National Environmental Health Survey (KoNEHS) 2012-2014. Int J Environ Res Public Health. (2021) 18:412. doi: 10.3390/ijerph18126412, PMID: 34199270 PMC8296250

[108] Yang C Li Y Ding R Xing H Wang R Zhang M. Lead exposure as a causative factor for metabolic associated fatty liver disease (MAFLD) and a lead exposure related nomogram for MAFLD prevalence. Front Public Health. (2022) 10:1000403. doi: 10.3389/fpubh.2022.1000403, PMID: 36311639 PMC9597460

[109] Frediani JK Naioti EA Vos MB Figueroa J Marsit CJ Welsh JA. Arsenic exposure and risk of nonalcoholic fatty liver disease (NAFLD) among U.S. adolescents and adults: an association modified by race/ethnicity, NHANES 2005-2014. Environ Health. (2018) 17:6. doi: 10.1186/s12940-017-0350-1, PMID: 29334960 PMC5769436

[110] Britton LJ Subramaniam VN Crawford DH. Iron and non-alcoholic fatty liver disease. World J Gastroenterol. (2016) 22:8112–22. doi: 10.3748/wjg.v22.i36.8112, PMID: 27688653 PMC5037080

[111] Sang H Lee KN Jung CH Han K Koh EH. Association between organochlorine pesticides and nonalcoholic fatty liver disease in the National Health and nutrition examination survey 2003-2004. Sci Rep. (2022) 12:11590. doi: 10.1038/s41598-022-15741-2, PMID: 35803990 PMC9270488

[112] Aronica L Ordovas JM Volkov A Lamb JJ Stone PM Minich D. Genetic biomarkers of metabolic detoxification for personalized lifestyle medicine. Nutrients. (2022) 14:768. doi: 10.3390/nu14040768, PMID: 35215417 PMC8876337

[113] Schoof EM Furtwängler B Üresin N Rapin N Savickas S Gentil C. Quantitative single-cell proteomics as a tool to characterize cellular hierarchies. Nat Commun. (2021) 12:3341. doi: 10.1038/s41467-021-23667-y, PMID: 34099695 PMC8185083

[114] Mansuri MS Williams K Nairn AC. Uncovering biology by single-cell proteomics. Commun Biol. (2023) 6:381. doi: 10.1038/s42003-023-04635-2, PMID: 37031277 PMC10082756

[115] Tajik M Baharfar M Donald WA. Single-cell mass spectrometry. Trends Biotechnol. (2022) 40:1374–92. doi: 10.1016/j.tibtech.2022.04.004 35562238

[116] Yamanishi K Maeda S Kuwahara-Otani S Watanabe Y Yoshida M Ikubo K. Interleukin-18-deficient mice develop dyslipidemia resulting in nonalcoholic fatty liver disease and steatohepatitis. Transl Res. (2016) 173:101–114.e7. doi: 10.1016/j.trsl.2016.03.010, PMID: 27063959

[117] Katsiki N Mikhailidis DP Mantzoros CS. Non-alcoholic fatty liver disease and dyslipidemia: an update. Metabolism. (2016) 65:1109–23. doi: 10.1016/j.metabol.2016.05.003, PMID: 27237577

[118] Truong T Kelly RT. What's new in single-cell proteomics. Curr Opin Biotechnol. (2024) 86:103077. doi: 10.1016/j.copbio.2024.103077, PMID: 38359605 PMC11068367

[119] Bennett HM Stephenson W Rose CM Darmanis S. Single-cell proteomics enabled by next-generation sequencing or mass spectrometry. Nat Methods. (2023) 20:363–74. doi: 10.1038/s41592-023-01791-5, PMID: 36864196

[120] Vanderaa C Gatto L. Replication of single-cell proteomics data reveals important computational challenges. Expert Rev Proteomics. (2021) 18:835–43. doi: 10.1080/14789450.2021.1988571, PMID: 34602016

[121] Yu SH Chen SC Wu PS Kuo PI Chen TA Lee HY. Quantification quality control emerges as a crucial factor to enhance single-cell proteomics data analysis. Mol Cell Proteomics. (2024) 23:100768. doi: 10.1016/j.mcpro.2024.100768, PMID: 38621647 PMC11103571

[122] Grégoire S Vanderaa C dit Ruys SP Kune C Mazzucchelli G Vertommen D. Standardized workflow for mass-spectrometry-based single-cell proteomics data processing and analysis using the scp package. Methods Mol Biol. (2024) 2817:177–220. doi: 10.1007/978-1-0716-3934-4_14, PMID: 38907155

[123] Ægidius HM Veidal SS Feigh M Hallenborg P Puglia M Pers TH. Multi-omics characterization of a diet-induced obese model of non-alcoholic steatohepatitis. Sci Rep. (2020) 10:1148. doi: 10.1038/s41598-020-58059-7, PMID: 31980690 PMC6981216

[124] Wernberg CW Ravnskjaer K Lauridsen MM Thiele M. The role of diagnostic biomarkers, omics strategies, and single-cell sequencing for nonalcoholic fatty liver disease in severely obese patients. J Clin Med. (2021) 10:930. doi: 10.3390/jcm10050930, PMID: 33804302 PMC7957539

[125] Ang MY Low TY Lee PY Wan Mohamad Nazarie WF Guryev V Jamal R. Proteogenomics: from next-generation sequencing (NGS) and mass spectrometry-based proteomics to precision medicine. Clin Chim Acta. (2019) 498:38–46. doi: 10.1016/j.cca.2019.08.010, PMID: 31421119

[126] Miller RM Jordan BT Mehlferber MM Jeffery ED Chatzipantsiou C Kaur S. Enhanced protein isoform characterization through long-read proteogenomics. Genome Biol. (2022) 23:69. doi: 10.1186/s13059-022-02624-y, PMID: 35241129 PMC8892804

[127] Reilly L Seddighi S Singleton AB Cookson MR Ward ME Qi YA. Variant biomarker discovery using mass spectrometry-based proteogenomics. Front Aging. (2023) 4:1191993. doi: 10.3389/fragi.2023.1191993, PMID: 37168844 PMC10165118

[128] Rajczewski AT Jagtap PD Griffin TJ. An overview of technologies for MS-based proteomics-centric multi-omics. Expert Rev Proteomics. (2022) 19:165–81. doi: 10.1080/14789450.2022.2070476, PMID: 35466851 PMC9613604

[129] Ng CKY Dazert E Boldanova T Coto-Llerena M Nuciforo S Ercan C. Integrative proteogenomic characterization of hepatocellular carcinoma across etiologies and stages. Nat Commun. (2022) 13:2436. doi: 10.1038/s41467-022-29960-8, PMID: 35508466 PMC9068765

[130] Peiseler M Schwabe R Hampe J Kubes P Heikenwälder M Tacke F. Immune mechanisms linking metabolic injury to inflammation and fibrosis in fatty liver disease - novel insights into cellular communication circuits. J Hepatol. (2022) 77:1136–60. doi: 10.1016/j.jhep.2022.06.012, PMID: 35750137

[131] Williams JK Anderson CM. Omics research ethics considerations. Nurs Outlook. (2018) 66:386–93. doi: 10.1016/j.outlook.2018.05.003, PMID: 30001880

[132] Vähäkangas K. Research ethics in the post-genomic era. Environ Mol Mutagen. (2013) 54:599–610. doi: 10.1002/em.21804, PMID: 23908016

[133] Letunica N van den Helm S McCafferty C Swaney E Cai T Attard C. Proteomics in thrombosis and hemostasis. Thromb Haemost. (2022) 122:1076–84. doi: 10.1055/a-1690-8897, PMID: 34753192

[134] Hanash S. Disease proteomics. Nature. (2003) 422:226–32. doi: 10.1038/nature01514, PMID: 12634796

[135] Manusov EG Diego VP Sheikh K Laston S Blangero J Williams-Blangero S. Non-alcoholic fatty liver disease and depression: evidence for genotype × environment interaction in Mexican Americans. Front Psych. (2022) 13:936052. doi: 10.3389/fpsyt.2022.936052, PMID: 35845438 PMC9283683

[136] Diego VP Manusov EG Almeida M Laston S Ortiz D Blangero J. Statistical genetic approaches to investigate genotype-by-environment interaction. *Rev Novel Extens Models* Genes. (2024) 15:547. doi: 10.3390/genes15050547, PMID: 38790175 PMC11121143

[137] Xu X Jin J Liu Y. Performance of FibroScan in grading steatosis and fibrosis in patients with nonalcoholic fatty liver disease: a meta-analysis. Arab J Gastroenterol. (2023) 24:189–97. doi: 10.1016/j.ajg.2023.08.003, PMID: 37996351

[138] Arvaniti P Giannoulis G Lygoura V Gatselis NK Gabeta S Rigopoulou E. FibroMeter scores are predictive noninvasive markers of advanced and significant liver fibrosis in patients with chronic viral hepatitis or metabolic dysfunction-associated steatotic liver disease. Ann Gastroenterol. (2023) 36:661–9. doi: 10.20524/aog.2023.0841, PMID: 38023979 PMC10662069

[139] Sanyal AJ Shankar SS Yates KP Bolognese J Daly E Dehn CA. Diagnostic performance of circulating biomarkers for non-alcoholic steatohepatitis. Nat Med. (2023) 29:2656–64. doi: 10.1038/s41591-023-02539-6, PMID: 37679433 PMC10579051

[140] Sangha K Chang ST Cheung R Deshpande VS. Cost-effectiveness of MRE versus VCTE in staging fibrosis for nonalcoholic fatty liver disease (NAFLD) patients with advanced fibrosis. Hepatology. (2023) 77:1702–11. doi: 10.1097/HEP.0000000000000262, PMID: 37018145

[141] Demirtaş D Ünal E İdilman İS Akçören Z Göktaş MA Boyraz MS. Magnetic resonance elastography in evaluation of liver fibrosis in children with chronic liver disease. Insights Imaging. (2023) 14:39. doi: 10.1186/s13244-023-01390-0, PMID: 36854936 PMC9975132

[142] Guan Y Yang J Xu Z. Comments on "metabolic dysfunction-associated fibrosis 5 (MAF-5) score predicts liver fibrosis risk and outcome in the general population with metabolic dysfunction". Gastroenterology. (2024) 167:1504–5. doi: 10.1053/j.gastro.2024.07.043, PMID: 39245408

[143] van Kleef LA Francque SM Prieto-Ortiz JE Sonneveld MJ Sanchez-Luque CB Prieto-Ortiz RG. Metabolic dysfunction-associated fibrosis 5 (MAF-5) score predicts liver fibrosis risk and outcome in the general population with metabolic dysfunction. Gastroenterology. (2024) 167:357–367.e9. doi: 10.1053/j.gastro.2024.03.017 38513745

[144] Bedogni G Bellentani S Miglioli L Masutti F Passalacqua M Castiglione A. The fatty liver index: a simple and accurate predictor of hepatic steatosis in the general population. BMC Gastroenterol. (2006) 6:33. doi: 10.1186/1471-230X-6-33, PMID: 17081293 PMC1636651

[145] Shah AG Lydecker A Murray K Tetri BN Contos MJ Sanyal AJ. Comparison of noninvasive markers of fibrosis in patients with nonalcoholic fatty liver disease. Clin Gastroenterol Hepatol. (2009) 7:1104–12. doi: 10.1016/j.cgh.2009.05.033, PMID: 19523535 PMC3079239

[146] Allen AM van Houten HK Sangaralingham LR Talwalkar JA McCoy RG. Healthcare cost and utilization in nonalcoholic fatty liver disease: real-world data from a large U.S. claims database. Hepatology. (2018) 68:2230–8. doi: 10.1002/hep.30094, PMID: 29774589 PMC6240503

